# Inside Out Integrin Activation Mediated by PIEZO1 Signaling in Erythroblasts

**DOI:** 10.3389/fphys.2020.00958

**Published:** 2020-07-31

**Authors:** Francesca Aglialoro, Naomi Hofsink, Menno Hofman, Nicole Brandhorst, Emile van den Akker

**Affiliations:** Sanquin Research and Landsteiner Laboratory, Department of Haematopoiesis, Amsterdam UMC, University of Amsterdam, Amsterdam, Netherlands

**Keywords:** PIEZO1, integrins, calcium signaling, integrin α4β1, erythroblastic island

## Abstract

The non-selective mechanosensitive ion channel PIEZO1 controls erythrocyte volume homeostasis. Different missense gain-of-function mutations in *PIEZO1* gene have been identified that cause Hereditary Xerocytosis (HX), a rare autosomal dominant haemolytic anemia. PIEZO1 expression is not limited to erythrocytes and expression levels are significantly higher in erythroid precursors, hinting to a role in erythropoiesis. During erythropoiesis, interactions between erythroblasts, central macrophages, and extracellular matrix within erythroblastic islands are important. Integrin α4β1 and α5β1 present on erythroblasts facilitate such interactions in erythroblastic islands. Here we found that chemical activation of PIEZO1 using Yoda1 leads to increased adhesion to VCAM1 and fibronectin in flowing conditions. Integrin α4, α5, and β1 blocking antibodies prevented this PIEZO1-induced adhesion suggesting inside-out activation of integrin on erythroblasts. Blocking the Ca^2+^ dependent Calpain and PKC pathways by using specific inhibitors also blocked increased erythroid adhesion to VCAM1 and fibronectins. Cleavage of Talin was observed as a result of Calpain and PKC activity. In conclusion, PIEZO1 activation results in inside-out integrin activation, facilitated by calcium-dependent activation of PKC and Calpain. The data introduces novel concepts in Ca^2+^ signaling during erythropoiesis with ramification on erythroblastic island homeostasis in health and disease like Hereditary Xerocytosis.

## Introduction

Human erythropoiesis occurs in the bone marrow in a specific niche called the erythroblastic island, composed of a central macrophage surrounded by differentiating erythrocyte precursors (Bessis, 1958; for review see Chasis and Mohandas, 2008; Manwani and Bieker, 2008; Heideveld et al., 2018). The interaction between the erythroblast and the macrophage is regulating erythroid maturation, proliferation, and survival (Manwani and Bieker, 2008). In addition, central macrophage phagocytes the extruded pyrenocyte (the extruded nucleus surrounded by a plasma membrane) left behind by the newly formed reticulocyte. Interactions between erythroid cells and macrophages are mediated by several adhesion molecules. During erythropoiesis, multiple adhesion molecules are expressed that mediate cell-cell and cell-extracellular matrix interactions, among which specific integrins (Chow et al., 2011; Palis, 2016; Seu et al., 2017). Integrin α4β1 [very late antigen 4 (VLA-4)] and α5β1 [very late antigen 5 (VLA-5)] expressed on erythroblasts can bind to counter-receptor vascular cell adhesion protein 1 (VCAM1, CD106) present on central macrophages or extracellular matrix (ECM) protein fibronectin, respectively (Sadahira et al., 1995; Chasis and Mohandas, 2008; Tanaka et al., 2009; Chow et al., 2013; Spring et al., 2013; Belay et al., 2017). Treatment with anti VLA-4 resulted in disruption of the island integrity *in vitro* and induces anemia in a mouse model and defects in stress erythropoiesis (Sadahira et al., 1995; Hamamura et al., 1996; Lee et al., 2006) clearly indicating the important role of these niche interactions. The activation state of integrins can be controlled by various processes including regulation via intracellular signaling, so called inside-out activation (Kim et al., 2003; Abram and Lowell, 2009; Hu and Luo, 2013) potentially providing a regulatory point to control erythroid-macrophage interactions. Inside-out activation of integrins is, among other mechanism, also controlled by intracellular Ca^2+^ concentration, with increased Ca^2+^ concentration leading to integrin activation (Jaconi et al., 1991; Schwartz et al., 1993; Shankar et al., 1993; Coppolino et al., 1997; Rowin et al., 1998; Bye et al., 2020; Shu et al., 2020). Several Ca^2+^ responsive proteins have been identified that control this inside-out integrin activation. For instance, the Ca^2+^ dependent Calpain unmasks the β-integrin binding motif of Talins leading to integrin activation (Goksoy et al., 2008; Mchugh et al., 2010). Inside out integrin activation is also mediated by Ca^2+^-dependent Protein kinase C (PKC; Kirchhofer et al., 1991; Harburger and Calderwood, 2009). One mechanism through which PKC operates is by activating the small GTPase Rap1 facilitating the formation of a complex with Rap1 effector Rap-1 interacting molecule (RIAM) and Talin, which causes integrin activation (Bos, 2005; Watanabe et al., 2008).

We and others have recently identified, a role for the non selective mechanosensitive cation channel, PIEZO1 during erythropoiesis (Caulier et al., 2020) as well as in *in vitro* cultured reticulocytes (Moura et al., 2019). Activation of PIEZO1 leads to Ca^2+^ influx and activation of Ca^2+^ dependent signal transduction among which NFATs, Calcineurin, MAPK, and calcium dependent PKCs (Von Lindern et al., 2000; Lanuti et al., 2006). Besides a role during erythropoiesis, PIEZO1 expression is maintained on erythrocytes where it regulates volume homeostasis. Indeed, activating mutations within PIEZO1 lead to dehydrated erythrocytes termed Hereditary Xerocytosis (HX; Andolfo et al., 2013; Bagriantsev et al., 2014; Cahalan et al., 2015). In endothelial cells activation of PIEZO1 has been associated with integrin activation and increased cell adhesion resulting in integrin-dependent focal adhesion kinase (FAK) signaling in flowing conditions (Albarran-Juarez et al., 2018). Concomitantly, reduced integrin activation was observed in an endothelium-specific PIEZO1 deficient mouse model (Albarran-Juarez et al., 2018). In agreement with this, depletion of PIEZO1 in small lung cancer cell lines also caused decreased integrin activation (Mchugh et al., 2012). PIEZO1 localization at focal adhesion induced integrin activation and FAK signaling regulated by the ECM (Chen et al., 2018). PIEZO1, located at the endoplasmic reticulum (ER), has been associated with integrin activation in epithelial cells (Mchugh et al., 2010). Inactivation of integrin β1 was observed in PIEZO1 siRNA knockdown in epithelial cells, resulting in reduced cell adhesion. Activation of Ca^2+^-protease Calpain and cleavage of its target Talin was demonstrated after PIEZO1 activation in epithelial cells (Mchugh et al., 2010). Here, we show that activation of PIEZO1 on erythroblasts using the selective agonist Yoda1 causes activation of downstream Ca^2+^-mediators Calpain and PKC, resulting in inside-out activation of integrins leading to increased adhesive properties of erythroblasts. The results give new insights into the regulation of integrin activation in erythroblasts, which will have consequences for erythroblastic island homeostasis.

## Materials and Methods

### Human Blood Sample

Human blood mononuclear cells were purified by density separation, following manufacturer’s protocol (GE Healthcare, Chicago, IL, United States). Informed consent was given in accordance with the Declaration of Helsinki, the Dutch National and Sanquin Internal Ethic Boards.

### Erythroblast Cell Culture and Differentiation

Erythroblasts were expanded as previously described (Heshusius et al., 2019). In short, cells were cultured and expanded in presence of EPO (2 UI/mL; ProSpec, East Brunswick, NJ, United States), human recombinant Stem Cell Factor (100 ng/mL, supernatant SCF producing cell line), and dexamethasone (1 μM; Sigma, St. Louis, MO, United States).

### Flow Adhesion Assay

Channels of the μ-Slide VI^0.4^ (Ibidi) were coated with 10 μg/ml recombinant human VCAM-1 (R&D systems, Minneapolis, MN, United States) or fibronectin (Sigma Aldrich, St. Louis, Missouri, United States) for 30 min at room temperature. The flow chambers were placed at room temperature on an inverted microscope. Tubes connected a syringe pump, containing CellQuin medium, with one side of the channel and the other side of the channel with a waste reservoir. Tubes and channels were washed with CellQuin medium at a flow speed of 7,5 ml/h. Samples consisted of 4 × 10^6^ erythroblasts and were incubated 10 min at 37°C with or without PKC inhibitor Gö6976 (Tocris, Bristol, United Kingdom), Calpain 1 and 2 inhibitor (Sigma), integrin α4 antibody (1 μg/ml, BD Biosciences, San Josè, CA, United States), β1 (0,5 μg/ml, Abcam, Cambridge, United Kingdom), and α5 (1 μg/ml, BD Biosciences, San Josè, CA, United States), GsMTx4 (Tocris, Bristol, United Kingdom), or Yoda1 (Sigma Aldrich, St. Louis, Missouri, United States). After incubation, samples were injected into the flow with a syringe and the pump was left running for 15 min unless otherwise stated. Nine pictures were taken across the channel after 15 min and attached erythroblasts were counted with ImageJ. Tubes were washed with water and fresh CellQuin medium in between samples. Data was analyzed with Prism 8 (GraphPad, San Diego, CA, United States). Unpaired student *t* test was used to calculate statistical significance between each condition. D’Agostino-Pearson test was used to assess the normality of the samples’ distribution.

### Flow Cytometry

Erythroblasts were treated with or without inhibitor, Yoda1, or Manganese (Mn^2+^). Samples were taken at different time points, each containing 200.000 erythroblasts in a volume of 200 μl, and were stained in a 96-well plate. The samples were kept on ice after treatment and during staining. Erythroblasts were incubated for 10 min at 37°C with or without PKC inhibitor Gö6976 (500 nM) or Calpain inhibitor 1 and 2 (1 mM) with gentle mixing. Samples were taken and resuspended into ice-cold PBS. Remaining erythroblasts were incubated at 37°C with or without Yoda1 or Manganese (II) chloride dihydrate solution in water (2 mM, Sigma Aldrich, St. Louis, MO, United States). Samples were taken after 10 and 30 min of incubation and were resuspended into ice-cold PBS. Staining was performed with 30 min incubation in FACS buffer (PBS, 0.5% BSA) with the following antibodies antiCD71 (Miltenyi Biotec, Bergisch Gladbach, Germany), antiCD235a OriGene Technologies, Inc., Rockville, MD, United States), antiCD49d (α4 integrin; BD Biosciences, San Jose, CA, United States), anti β1 integrin unconjugated (Abcam, Cambridge, United Kingdom). Staining was followed by two washing steps with FACS buffer, followed or not by staining with secondary antibody. After the last staining and wash, the cells were resuspended into 200 μl of FACS buffer. Cells were transferred to FACS tubes before measurement on FACSCanto II (BD Biosciences, San Josè, CA, United States). Data was analyzed with FlowJo^®^ software (BD Biosciences, San Josè, CA, United States) and Prism 8 (GraphPad, San Diego, CA, United States).

### Calpain Activity Assay

Calpain activity was measured with the Calpain Activity Fluorometric Assay Kit (Sigma Aldrich, St. Louis, MO, United States), following manufacturer protocol. Samples were measured in triplicates. In short, the amount of 2 × 10^6^ erythroblasts was taken per sample. Samples were incubated with or without Yoda1 (1 μM or 5 μM, 10 min, 37°C). After centrifugation, samples were washed in PBS and resuspended into 100 μl Extraction Buffer. Samples were incubated (20 min, on ice) and spun down (10.000 *g*, 1 min). Cell lysate was transferred to a new tube and kept on ice. 10x Reaction buffer and Calpain substrate was added to 85 μl cell lysate and samples were transferred to 96-well plate. After incubation in the dark (1 h, 37°C), samples were measured with 400 nm excitation filter and 505 nm emission filter with a plate reader (BioTek, Winooski, VT, United States). Positive control (1 μl Active Calpain) and negative control (1 μl Calpain inhibitor) were taken along. Data was analyzed with Prism 8. Two-way ANOVA was used to calculate statistical significance between samples.

### Western Blot

Cells were lysed in CARIN lysis buffer (20 mM Tris–HCl pH 8.0, 138 mM NaCl, 10 mM EDTA, 100 mM NaF, 1% Nonidet P-40, and 10% glycerol). Following Bradford protein quantification (Bio-Rad Laboratories, Hercules, CA, United States), lysates were boiled in Laemmli sample buffer [2% sodium dodecyl sulfate (SDS) wt/vol, 10% glycerol, 5%2-mercaptoethanol, 60 mM Tris–HCl pH6.8, and trace amount brome-phenol blue; 3 min, 95°C], subjected to SDS-polyacrylamide gel electrophoresis, blotted using iBlot-PVDF blotting system (Thermo Fisher Scientific, Bleiswijk, Netherlands), and stained as indicated in the figure legends.

### Fluorescent Microscopy Imaging

Expression of integrin β1 on erythroblasts was determined with live imaging. Erythroblasts, 25 × 10^5^ in 0.5 ml CellQuin, were incubated with integrin β1 antibody [1:100, clone (P5D2), Abcam, Cambridge, United Kingdom, Alexa Fluor 488], DRAQ5^TM^ (1:2500, Abcam, Cambridge, United Kingdom), and CD235a (Glycophorin A, 1:200, PB, Miltenyi, Bergisch Gladbach, Germany). Microscopy images were taken with an Axiovert 200 microscope (Zeiss, Oberkochen, Germany) with bright field, DAPI, Alexa Fluor 488, and Alexa Fluor 647 filters using 40x/1.3 oil objective. Software ZEN2.3 (Zeiss, Oberkochen, Germany) was used to analyze and convert images.

## Results

### Integrin Subunits Are Differently Expressed in Erythroblasts

**FIGURE 1:**
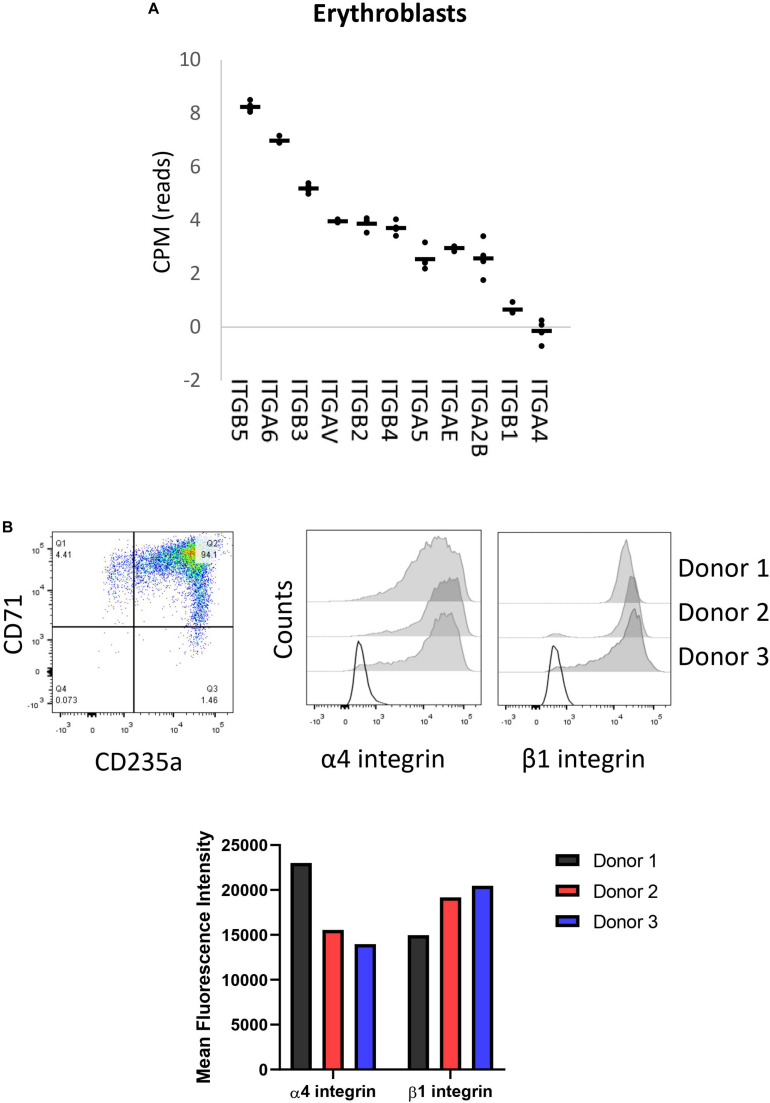
Erythroblasts integrin expression. **(A)** Integrin mRNA expression (count per million) in CD71+/CD235a+ erythroblasts; data mined from Heshusius et al. (2019). Gene expression was found for 11 integrin subunits: six α sub-units (*ITGA*) and five β sub-units (*ITGB*; *n* = 4). **(B)** Dot plot showing a representative CD71/CD235 expression plot depicting the erythroblast stage of analysis. Right histograms and bar graph indicate expression of α4 integrin and β1 integrin in CD71+CD235a+ erythroblasts in 3 donors as measured by flow cytometry.

We have previously reported membrane expression of ITGA4 and ITGB1 (VLA-4) in our *in vitro* cultured erythroid cells (Heideveld et al., 2018). To further evaluate the expression of integrin subunits present on erythroblasts, we data-mined RNA-sequencing that we reported previously containing RNA-expression profiles of CD71+/CD235+ erythroblasts (Heshusius et al., 2019). This analysis showed mRNA expression of eleven integrin subunits: six α subunits (*ITGA*) and five β subunits (*ITGB*; Figure 1A). At the erythroblast stage mRNA expression of integrin subunits α2B (*ITGA2B*), α4 (*ITGA4*), α5 (*ITGA5*), αE (*ITGAE*), αV (*ITGAV*), α6 (ITGA6), and subunits β1 (*ITGB1*), β2 (*ITGB2*), β3 (*ITGB3*), and β4 (*ITGB4*) were observed albeit with significantly different expression levels (Figure 1A). At the stage where erythroblast are CD71+/CD235+ (Figure 1B), α4 and β1 RNAs are most abundantly expressed followed by *ITGAIIB*, *ITGAE*, *ITGA5, ITGB4*, while *ITGAV, ITGB3, ITGB2, and ITG6* are lowly expressed. This confirms the presence of α4β1 (VLA-4; Heideveld et al., 2018) and would further allow α5β1 (VLA-5) and low expression of αVβ1, α6β1, αIIbβ3 (GPIIb/IIIa), αVβ3 (CD61), and αVβ5. Integrins αIIbβ3 (GPIIb/IIIa) and αVβ3 (CD61) are associated with megakaryopoiesis and platelet homeostasis and expression of the beta partner ITGB3 (β3) is significantly downregulated during differentiation (Heshusius et al., 2019). Of note, no interaction partners for the subunits ITGAE, ITGB4, and ITGB2 were detected (Supplementary Figure 1A). Expression of the most abundant integrin subunits α4, and β1 was confirmed by flow cytometry in three donors (Figure 1B and Supplementary Figure 1B) and imaging (Figure 1B and Supplementary Figure 1C).

### Activation of PIEZO1 With Yoda1 Leads to Integrin Activation and Increases Erythroblasts Adhesion

**FIGURE 2:**
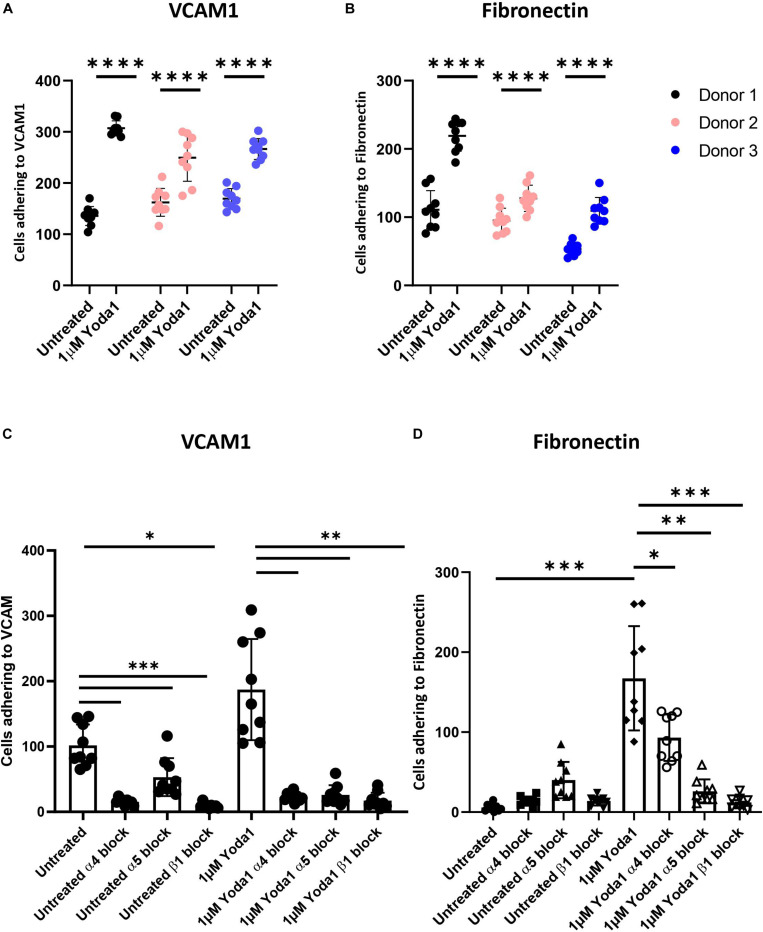
Increased erythroblast binding to VCAM-1 and fibronectin after Yoda1 mediated PIEZO1 stimulation. **(A,B)** Erythroblasts were treated or not with 1 μM of Yoda1 for 10 min and subjected to a flow assay as described in Materials and Methods (7.5 ml/h flow rate). Adhesion of erythroblasts to VCAM **(A)** or fibronectin **(B)** was quantified using imageJ (*n* = 9 pictures of different donors; data depicted as mean ± SD; ^* * **^P < 0.0001; **C,D**) Erythroblasts were pre-treated with anti-α4, anti-α5, or anti-β1 and stimulated or not with 1 μM Yoda1 (10 min) as indicated and flowed over a surface coated with VCAM **(C)** or fibronectin (**D**; data depicted as mean ± SD mean *n* = 9 images was used to calculate statistic; ^∗^*P* < 0.05, ^∗∗^*P* < 0.01, ^∗∗∗^*P* < 0.001, and ^* * **^P < 0.0001).

Activation of mechanosensitive PIEZO1 has been shown to lead to inside out integrin activation on non-hematopoietic cells and specific Ca^2+^ dependent signal transduction in erythroblasts (Rowin et al., 1998; Mchugh et al., 2010; Caulier et al., 2020). The high abundant integrin subunit β1 in combination with α4 (VLA-4) and α5 (VLA-5) on erythroblasts can bind VCAM1 and fibronectin, respectively. The effect of chemical activation of PIEZO1 on integrin activation and adhesion of CD71HighCD235aHigh erythroblasts to VCAM-1 and fibronectin was assessed. Erythroblasts were activated by 2 mM Mn^2+^, a strong integrin activator, or with different concentration of the PIEZO1 agonist Yoda1 in presence of soluble VCAM1. Compared to untreated, incubation with Mn^2+^ or Yoda1 increased binding to anti-VCAM1 antibody, albeit that activation with Yoda1 was lower compared to Mn^2+^ (Supplementary Figure 2A). Note that the expression of total integrin β1 remained unchanged throughout the different treatments (Supplementary Figure 2B). To assess the adhesive properties of erythroblasts, a flow adhesion assay in which the erythroblasts are flowed within a flow cell that is coated with VCAM1 or fibronectin was used. Increased adhesion to VCAM1 (Figure 2A) and fibronectin (Figure 2B) was observed when erythroblasts were incubated with the PIEZO1 agonist Yoda1 (Supplementary Figure 3A). Of note, a Yoda1 concentration of 1 μM was chosen as short term and long term use of higher concentrations have been shown to be detrimental, both in terms of adhesion to VCAM1 (Supplementary Figure 3B), as well as cell viability, respectively (Supplementary Figure 4). Of note, different flow speeds did not influence the increased adhesion in Yoda1 treated cells (Supplementary Figure 3C). Pre-incubation with an antagonizing β1 antibody thus blocking the common beta integrin subunit within VLA-4 and VLA5 results in a significant reduction of both Yoda1-induced and steady state adhesion to VCAM1 or fibronectin (Figures 2C,D). Blocking VLA-4 or VLA-5 specifically using anti-α4 or anti-α5 blocking antibodies, respectively, also led to decreased adhesion (Figures 2C,D). Note that blocking VLA-4 using α4-integrin antibodies leads to lower inhibition of adhesion to fibronectin compared to blockage of VLA-5, which has been shown before for mouse erythroblasts (Eshghi et al., 2007). Treatment with GsMTx4, a mechanosensitive ion channel inhibitor, resulted in decreased adhesion following Yoda1 treatment but did not revert the basal adhesion of untreated cells (Supplementary Figure 5). In conclusion, the results indicate that erythroblast adhesion to VCAM1 and fibronectin is integrin-dependent and can be increased by activating PIEZO1.

### Activation of PIEZO1 Leads to Increased Calpain Activity

**FIGURE 3:**
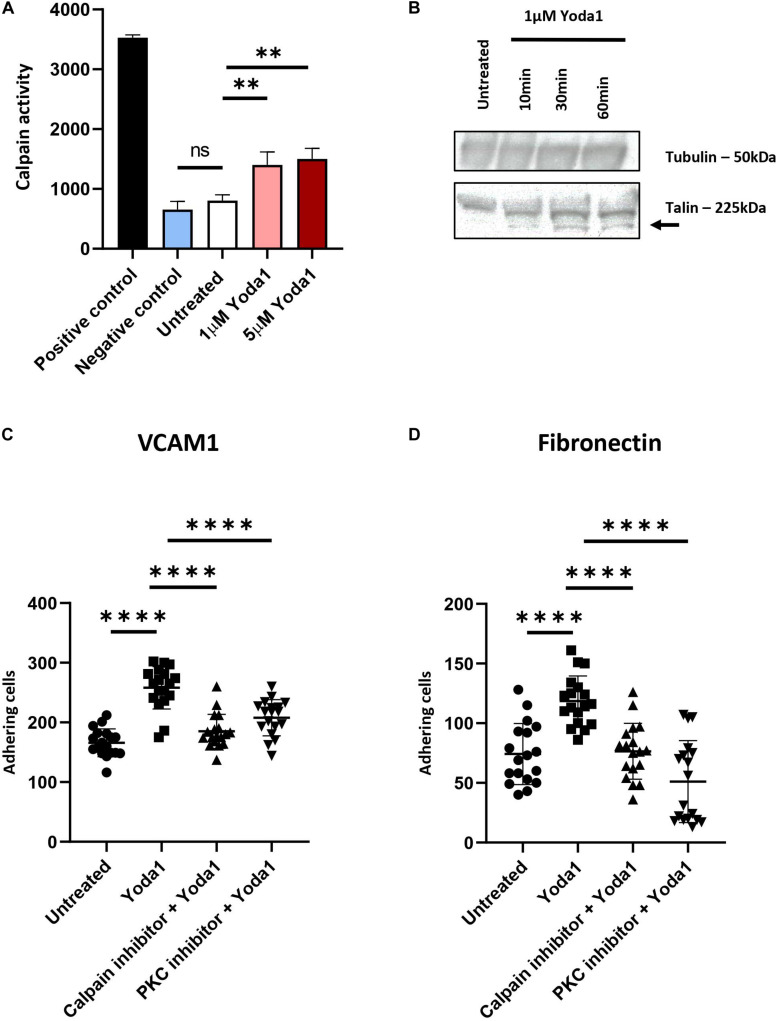
PIEZO1 stimulation with Yoda1 results calpain and PKC dependent integrin activation. **(A)** In untreated and Yoda1 treated erythroblasts, the calpain activity assay measured the absorbance of cleaved calpain substrate at 505 nm, which is quantified using a fluorescence plate reader. Active calpain and inhibited calpain where used as positive and negative controls, respectively (*n* = 3, ^∗∗^*P* < 0.01). **(B)** Expression of Talin (±225 kDa) and the cleavage of Talin (±190 kDa; indicated by arrow) in untreated erythroblasts and erythroblasts incubated with 1 μM Yoda1 for 10, 30, and 60 min. The same amount of protein was loaded and Tubulin (±50 kDa) was used as loading control (representative of *n* = 2). **(C,D)** Erythroblasts were pretreated with PKC inhibitor (500 nM) and calpain inhibitor (1 mM) and stimulated with and without 1 μM Yoda1 (10 min) as indicated and flowed over a surface coated with VCAM **(C)** or fibronectin (**D**; *n* = 2 donors; data depicted as mean ± SD; mean of *n* = 18 images (of two different donors) was used to calculate statistic ^* * **^P < 0.0001).

Ca^2+^ influx caused by PIEZO1 activation has been shown in endothelial cells, erythroid cells and erythrocytes to lead to activation of calcium dependent pathways, among which the positive regulators of integrin activation calpain, PKC and Talin (Bos, 2005; Watanabe et al., 2008; Mchugh et al., 2010). To demonstrate whether calpain is activated following PIEZO1 activation, cells were treated with 1 μM Yoda1 or 5 μM Yoda1 for 60 min or left untreated. Calpain activity was significantly increased upon Yoda1 treatment compared to untreated cells. No significant difference between 1 and 5 μM Yoda1 was observed suggesting that 1 μM Yoda1 is sufficient to activate the pool of calpain within erythroblasts. Of note, short term treatment with 1 μM Yoda1 or 5 μM Yoda1 did not influence cell viability (Supplementary Figure 4). Note that Calpain activity is similar between untreated controls and samples treated with a calpain inhibitor 1 and 2, indicating that in erythroblasts basal calpain activity is low (Figure 3A). Calpain cleaves Talin to an activated state mediating inside-out integrin activation (Goksoy et al., 2008; Mchugh et al., 2010). Note that 1 μM Yoda1 led to a modest but clear increase in cleaved activated Talin, depicted in western blot as a lower molecular weight migrating band (Figure 3B).

### Flow-Induced Adhesion to VCAM1 and Fibronectin Is Dependent on PKC and Calpain

Inside-out integrin activation can be mediated by several signaling cascades among which calpain and PKC-dependent pathways. Ca^2+^ influx through PIEZO1 activation by Yoda1, or in patient-derived erythroblasts with specific activation mutations in PIEZO1, causes activation of PKC-dependent signal transduction that can be partly blocked by inhibiting calcium-dependent PKCs (Caulier et al., 2020). Together with the observation that Calpain is activated after PIEZO1 activation we evaluated whether the increased adhesion is a consequence of Calpain and/or PKC activity leading to integrin activation in Yoda1 treated erythroblasts. Pretreatment with Calpain inhibitor or Gö9676 (an inhibitor of Ca^2+^ dependent PKCs) before Yoda1 incubation decreased adhesion of Yoda1 treated erythroblasts to VCAM1 (Figure 3C) and fibronectin (Figure 3B) to untreated background adhesion levels. Of note, inhibition of the PKC or Calpain pathway does not lead to complete block of adhesion as observed upon blocking integrin subunits (Figures 3C,D). The data indicates that PIEZO1 activation leads to both PKC and Calpain dependent integrin activation. Note that the inhibition of adhesion is primarily affecting the Yoda1 mediated increase in adhesion. This is in contrast to blocking specific integrin subunits, which also blocks the basal level of erythroblast adhesion. This is in agreement with the low level of calpain activation in steady state erythroblasts (Figure 3A).

## Discussion

Ca^2+^ influx has been linked to integrin activation in several cell types (Jaconi et al., 1991; Schwartz et al., 1993; Shankar et al., 1993; Coppolino et al., 1997; Rowin et al., 1998; Bye et al., 2020; Shu et al., 2020). This report identifies for the first time a role of the mechanosensor PIEZO1 in integrin activation in erythroblasts. We found PIEZO1 activation leads to inside-out VLA4 and VLA5 activation in erythroblasts. This activation is dependent on Ca^2+^ activated PKCs and calpain. Together, the data indicates that erythroid cells can perceive mechanical forces and in response to this activate VLA5 and VLA4. This may have consequences for bone marrow retention and migration, for instance with the central macrophage within the erythroid island, which expresses VCAM1, the ligand of VLA4.

The semi-solid bone marrow is occupied with a heterogeneous pool of cells that will perceive and induce particular cellular programs upon shear forces from fluid movements, tensile strain, hydrostatic pressure and other physical forces (Meyer et al., 2016; Kim and Bixel, 2020). The consequences of these forces on haematopoiesis are beginning to emerge. For instance, it has been shown that hydrostatic pressure improves the clonogenic potential and CD34+ cell number in ex-vivo cultures (Kim and Bixel, 2020). PIEZO1 may be one of the sensors that could be involved in perceiving these forces and integrating them into a cellular response, for instance through integrin activation as shown here. Indeed, PIEZO1 has been shown to mediate tissue hydrostatic pressure sensing in various hematopoietic cells including T-cells and monocytes (Solis et al., 2019). More specific to the erythroid system, adhesive interactions facilitated by integrins between (i) the central macrophage and developing erythroblasts, (ii) the erythroblasts themselves, and (iii) the erythroblasts and ECM are important for the support and regulation of erythropoiesis (Vuillet-Gaugler et al., 1990; Hanspal, 1997; Arroyo et al., 1999). Loss of α4 integrin affects erythropoiesis in the mice fetal liver and bone marrow, in terms of decreased cellularity. Moreover, in an *in vitro* system, *ITGA4* null erythroid progenitors display defective proliferation and migration (Arroyo et al., 1999). Ulyanova et al. (2011) observed that *ITGA4*-deficient mice are defective in triggering an optimal stress erythropoiesis response with a decrease in peripheral blood reticulocytes and inability to increase haematocrit after treatment with Phenylhydrazine. The effect was less evident in *ITGA5* deficient mice (Ulyanova et al., 2014). Indeed, integrins have been implicated in regulating the proliferative response of erythroid cells through inhibition of apoptosis by upregulating BCL-XL (Eshghi et al., 2007). Activation of VLA4 and VLA5 via PIEZO1 mechanoactivation in the bone marrow may thus strengthen anti-apoptotic signaling pathways. Interestingly, deletion of α4 integrin caused egress of erythroid cells from the bone marrow during erythroid maturation at homeostasis and during stress erythropoiesis (Ulyanova et al., 2014). This suggests that VLA4 plays a role in maintaining erythroid cells within the bone marrow, which may be strengthened upon PIEZO1-induced VLA4 activation.

The role of PIEZO1 during erythropoiesis has been investigated only recently. Increased activation of PIEZO1 was followed by activation of specific (Ca^2+^ dependent) pathways including NFAT and MAPK (Caulier et al., 2020). We found involvement of the PIEZO1-induced Ca^2+^ dependent Calpain and PKC activation in erythroblasts, which were both essential for PIEZO1-induced VLA4 and VLA5 activation. Although we found increased proteolysis of the calpain target Talin upon Yoda1 treatment, more research needs to be done to identify the intermediate steps between calpain, PKC activation and VLA4/VLA5 activation. We found that erythroblast display a basal level of adhesion toward VCAM1 and fibronectin in flow adhesion assays. Blocking α4-integrin or α5-integrin resulted in complete block of adhesion, including the basal adhesion indicating the complete dependence on VLA4 and VLA5 for adhesion to VCAM and Fibronectin. Of note, untreated cells adhered in the same manner independently of the flow speed used, supporting a basal adhesion at steady state (Supplementary Figure 3C). We find that erythroblasts treated with GsMTx4 inhibited the Yoda1-induced adhesion of erythroblasts. However, it must be noted that GsMTx4 is a general inhibitor of mechanosensing by cells and not specific for PIEZO1, as it incorporates itself into the lipid layer, and allows for partial relaxation upon mechanical stress (Gnanasambandam et al., 2017). Nevertheless, the experiments show that integrin activation is dependent on mechanosensing. Interestingly, inhibiting PKC, or Calpain reduced adhesion to basal levels but did not fully block adhesion as observed upon using anti- α4-integrin or α5-integrin. This may suggest that the basal activation of VLA4 and VLA5 is dependent on other PKC and calpain independent processes. Indeed, inhibition of calpain does not significantly decrease the total calpain activity in erythroblasts suggesting that basal levels of calpain activity in steady state erythroblasts is low. In conclusion, we show PIEZO1-induced inside-out integrin activation, facilitated by Ca^2+^-dependent activation of PKC and Calpain. This knowledge could be of crucial importance in the investigation of the role of Ca^2+^ during erythropoiesis, and in particular of the dynamics of the erythroblastic island, both in health and disease (such as HX). Such a condition could lead to stress erythropoiesis (which would explain the reticulocytosis observed in these patients), a situation where support of the erythroblastic island microenvironment has been shown to be important.

## Data Availability Statement

All datasets presented in this study are included in the article/Supplementary Material.

## Ethics Statement

The studies involving human participants were reviewed and approved by Dutch National and Sanquin Internal Ethic Boards. The patients/participants provided their written informed consent to participate in this study.

## Author Contributions

FA designed the experimental setup, performed and designed the experiments, and wrote the manuscript. NH, MH, and NB designed and performed the specific experiments. EA designed the experimental setup, and supervised and edited the manuscript. All authors contributed to the article and approved the submitted version.

## Conflict of Interest

The authors declare that the research was conducted in the absence of any commercial or financial relationships that could be construed as a potential conflict of interest.
