# Supplementary material for: Inside Out Integrin Activation Mediated by PIEZO1 Signaling in Erythroblasts
Source: Front Physiol. 2020 Jul 31;11:958. doi: 10.3389/fphys.2020.00958 (PMC7411472; doi:10.3389/fphys.2020.00958)
Supplement: Supplementary file 1 [file Data_Sheet_1.PDF]

# Supplemental figure 1

A

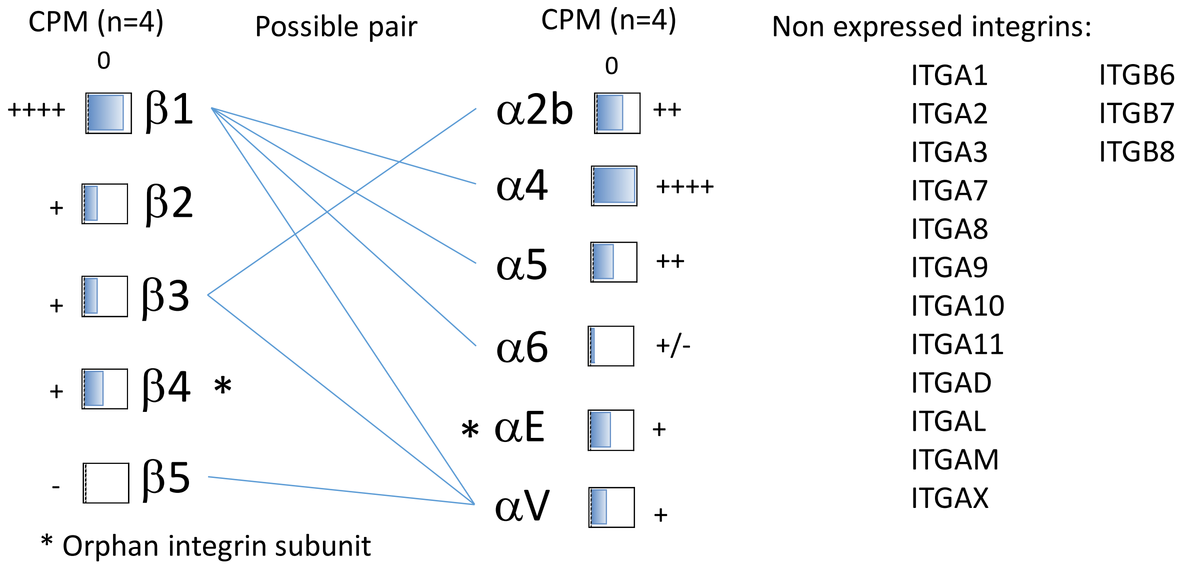


* Orphan integrin subunit

\+ and - expression in non-hemoglobinized CD71+CD235low erythroblasts

B

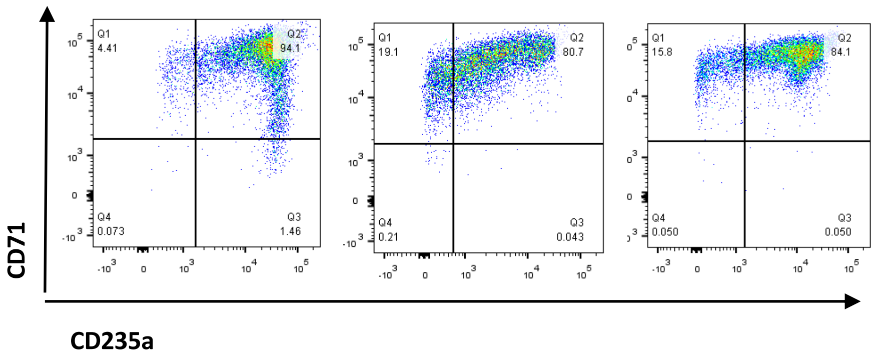


C

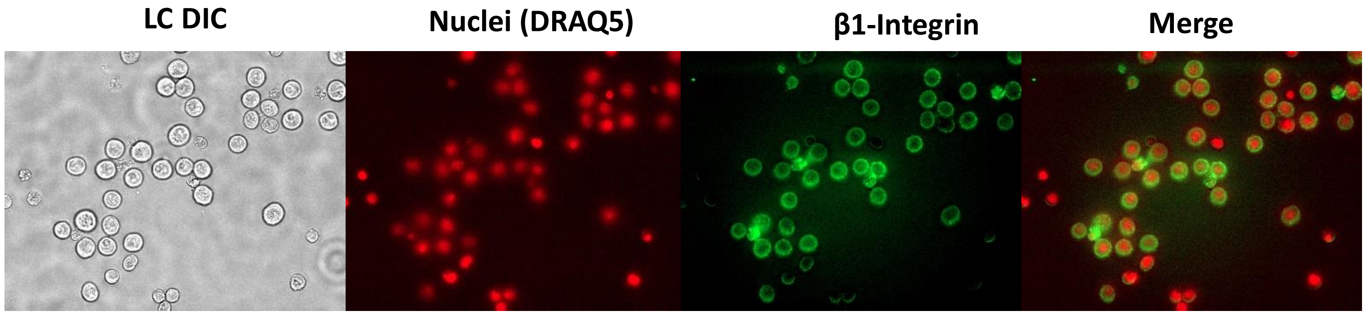

# Supplemental figure 2

A

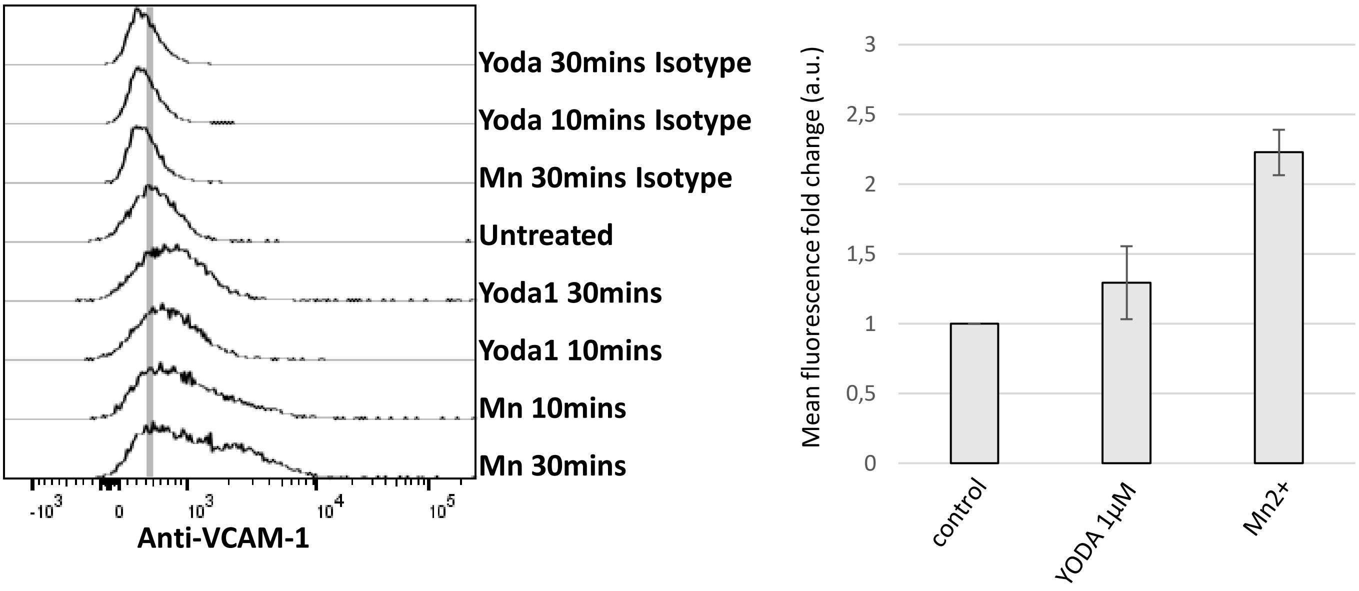


B

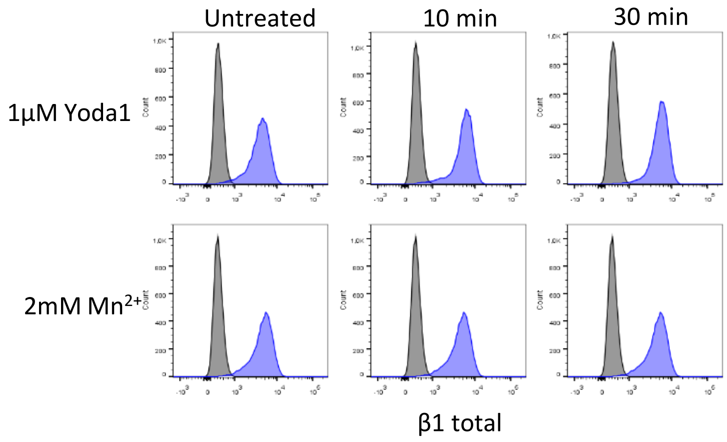


C

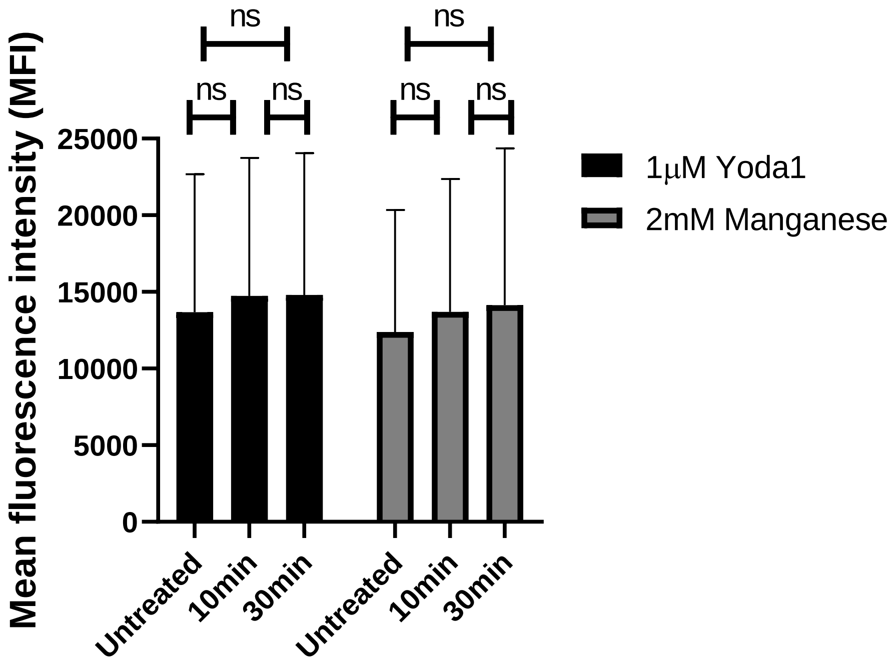

Supplemental figure 3

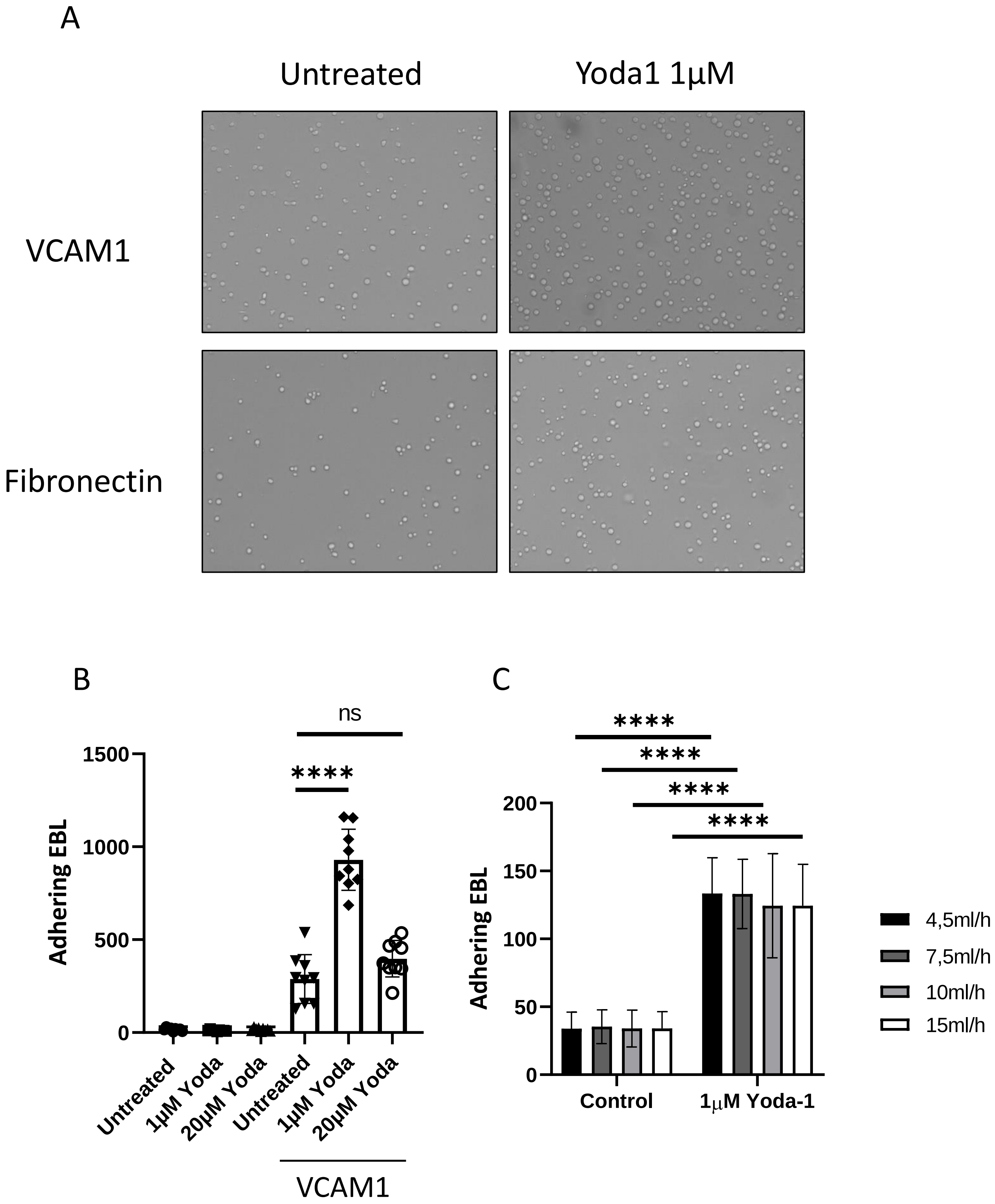

Supplemental figure 4

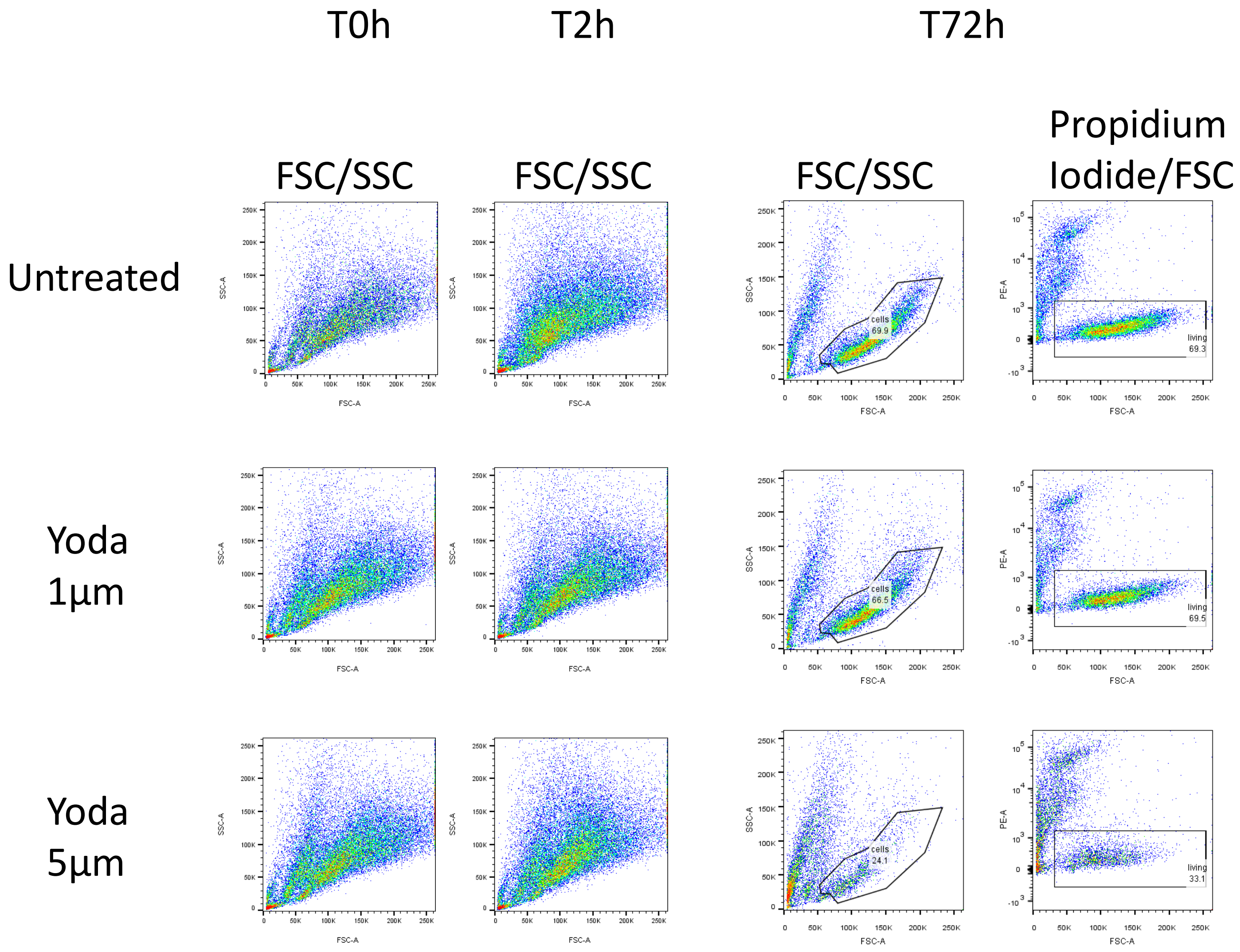

Supplemental figure 5

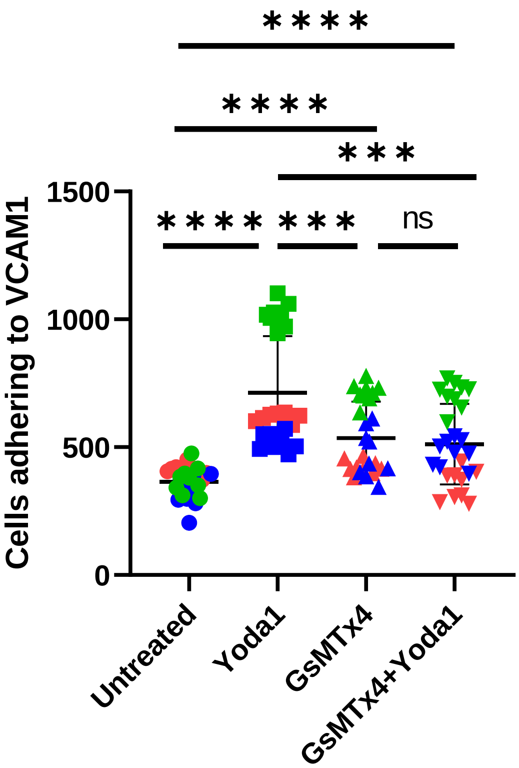

**Supplemental Figure 1 - Integrin expression in erythroblasts. (A)** Plot showing the possible interactions of integrin chains as expressed in erythroblasts. The blue graphs represent the RNA expression level in counts per million with overall expression levels indicated with a + and/or a -. *=orphan integrin subunits. Non-expressed integrins indicate integrins for which no RNA was detected in erythroblasts. **(B)** Dot plot showing the expression of CD71+CD235a+ on erythroblasts from 3 donors measured by flow cytometry and belonging to figure 1B. **(C)** Expression of integrin β1 was assessed with fluorescence microscopy. β1 integrin (green) is distributed over erythroblasts membrane. DNA staining (red) was performed using DRAQ5.

**Supplemental Figure 2 - Integrin activation following PIEZO1 activation results in increase in VCAM1 binding detectable by flow cytometry. (A)** Incubation with VCAM1 and activation of PIEZO1 by Yoda1 results into increase in VCAM-1 binding. Manganese (Mn) was used as a positive control for integrin activation (Bar graph depicting Mean Fluorescence Intensity of 2 pulled experiments). **(B)** Yoda1 and Manganese treatments do not influence expression of integrin β1. Erythroblasts were treated with Yoda1 at 1µM concentration, and 2mM Manganese over the course of 10 and 30 minutes, and expression of integrin β1 was assessed in flow cytometry. **(C)** Representation of β1 expression in three donors (ns= not significant).

**Supplemental Figure 3 - Adhesion of erythroblasts to VCAM1 and fibronectin under different conditions. (A)** Representative pictures of erythroblasts adhering to VCAM1 or fibronectin. Erythroblasts were stimulated with and without 1µM Yoda1 (10 min) as indicated and flowed over a surface coated with VCAM or fibronectin **(B)** Adhesion of erythroblasts to VCAM1 following treatment with Yoda1 at 1µM and 20µM concentrations (average of 9 pictures taken). Erythroblasts were stimulated with and without 1 or 20µM Yoda1 (10 min) as indicated and flowed over a surface coated with VCAM (ns=not significant, **** P<0.0001) **(C)** Adhesion of erythroblasts to VCAM1 at different flow speeds. Erythroblasts were stimulated with and without 1µM Yoda1 (10 min) as indicated and flowed over a surface coated with VCAM at 4.5, 7.5, 10 and 15 ml/h flow speed (Pull 2 donors, average of 9 pictures taken; **** P<0.0001).

**Supplemental Figure 4 – Treatment of erythroblasts with 1µM Yoda1 or 5µM Yoda1 does not influence viability.** Dot plot depicting FSC/SSC and Propidium Iodide/SSC of erythroblasts treated with 1µM Yoda1 or 5µM Yoda1 or left untreated, at timepoint 0h, 2h and 72h.

**Supplemental Figure 5 – Treatment with GsMTx4 induces decrease in adhesion following Yoda1 treatment.** Representative pictures of erythroblasts adhering to VCAM1. Erythroblasts were pretreated with 2.5µM of GsMtx4 for 30min and stimulated with and without 1µM Yoda1 (10 min) as indicated and flowed over a surface coated with VCAM (different colors indicate different donors, **** P<0.0001, *** P<0.001).
